# Cerebrospinal Fluid Biomarkers in Multiple System Atrophy Relative to Parkinson's Disease: A Meta-Analysis

**DOI:** 10.1155/2021/5559383

**Published:** 2021-05-31

**Authors:** Dan Xie, Ling Feng, Hongyan Huang, Quanzhen Zhao, Pingping Ning, Qiuyan Shen, Haitao Lu, Fang Xu, Yanming Xu

**Affiliations:** Department of Neurology, Sichuan University West China Hospital, Chengdu, 610041 Sichuan, China

## Abstract

**Objective:**

To investigate the differences of candidate cerebrospinal fluid (CSF) biomarkers associated with multiple system atrophy (MSA) and Parkinson's disease (PD).

**Method:**

Here, a systematic review and meta-analysis were conducted on studies related to CSF biomarkers associated with MSA and PD obtained from PubMed, Web of Science, Embase, and Cochrane databases. Data were pooled where appropriate and used to calculate standardized mean differences (SMDs) with 95% confidence intervals (CI). Heterogeneity was assessed using the *I*^2^ statistic while Egger's test was used to test for existing publication bias.

**Results:**

MSA patients had higher CSF t-tau (SMD = 0.41, 95% CI: 0.10 to 0.72) and YKL-40 (SMD = 0.63, 95% CI 0.12 to1.15) as well as DJ-1 (SMD = 1.05, 95% CI 0.67 to 1.42) levels than PD patients, while CSF p-tau (SMD = −0.17, 95% CI, -0.31 to -0.02) and A*β*-42 (SMD = −0.33, 95% CI, -0.55 to -0.12) levels in MSA patients were lower than those in PD patients. There were no differences in CSF's GFAP and Flt3 ligand levels in both MSA and PD patients.

**Conclusion:**

The study revealed the differences in CSF biomarker levels between MSA and PD cohorts that can be further explored to clinically distinguish MSA from PD.

## 1. Introduction

Multiple system atrophy (MSA) is a progressive neurodegenerative disease whose prevalence ranges between 3.4 and 4.9 persons in every 100,000 people. Its prevalence increases to 7.8 persons in every 100,000 people among persons with more than 40 years [1]. It is characterized by autonomic failures such as urinary disorders, orthostatic hypotension, and erectile dysfunction in men and parkinsonian features as well as cerebellar and pyramidal features in varying combinations [1]. Currently, only symptomatic therapies that include pharmacologic and nonpharmacologic approaches are available to treat patients with MSA [1].

According to current diagnostic guidelines of MSA, clinically probable and possible diagnosis is mainly based on clinical and neuroimaging features [2]. However, the accuracy of the clinical diagnosis of MSA is still unsatisfactory. Osaki et al. reported that the sensitivity of clinically possible and probable MSA diagnosis was only 41% and 18%, respectively, at the first clinic visit [3]. Koga et al. pathologically confirmed that 62% of patients who were clinically diagnosed with MSA met the pathologic criteria for MSA [4]. MSA is often confused with Parkinson's disease (PD). Both diseases overlap largely in clinical manifestations, especially at the early stage. However, they differ in their management and prognosis thus making it very important to distinguish the two [5]. Early diagnosis is essential to predict the clinical course accurately, avoid a delay in the proper treatment, and thus preserve the motor function [6]. Accurate diagnosis of patients recruited in clinical trials is also critical. It is therefore important to urgently explore reliable methods for the early diagnosis of the disease.

The pathologic hallmark of MSA is *α*-synuclein aggregation mainly in the glial cytoplasmic inclusions (GCIs) [1]. Formation of toxic GCIs interferes with neuronal support thus resulting in neuronal dysfunction such as neuroinflammation and mitochondrial dysfunction. This significantly contributes to reactive astrogliosis and neuronal loss [1, 7]. Besides *α*-synuclein, other proteins such as ubiquitin and Parkinson disease protein 7 (DJ-1) are also involved in the neurodegenerative process [7]. Cerebrospinal fluid (CSF) directly contacts the brain. Cognizant of this, biological and molecular processes occurring in the brain can be reflected by protein/peptide changes in CSF. CSF can be a reliable biomarker source that improves diagnostic accuracy and monitors the pathogenic progression of disease [8]. Biomarkers have been proven to improve the diagnostic accuracy in some neurodegenerative diseases such as Alzheimer's disease [9]. CSF biomarkers can also provide clues to identify the pathophysiological pathways involved in disease progression. Moreover, they can also help in the development of potential targets for disease-modifying treatments [10]. As such, exploring CSF biomarkers of MSA is significant.

Numerous studies have attempted to identify biomarkers that can differentiate MSA and PD. The studies have mainly focused on proteins involved in disease-related pathology, axonal degeneration, oxidative stress, and neuroinflammation [11]. Despite this, there are substantial discrepancies among the published studies and no biomarker is currently available [11]. Several meta-analysis studies have revealed the differences of CSF total *α*-synuclein concentration between patients with MSA and PD, though the results among these studies are not consistent [12–14]. And several meta-analysis are already done showing that neurofilament light chain protein (NF-L) concentrations in CSF are significantly increased in MSA and progressive supranuclear palsy (PSP) compared to PD [15–17]. To date, no systematic synthesis of differences in CSF biomarkers between MSA and PD has been conducted. Herein, a meta-analysis study was conducted to examine the discrepancies in the levels of various CSF biomarkers. The study was aimed at providing more evidence for differential diagnosis of MSA and PD.

## 2. Materials and Methods

### 2.1. Search Strategy

Recommendations made by the Meta-Analysis of Observational Studies in Epidemiology Group, the Preferred Reporting Items for Systematic Reviews and Meta-Analyses (PRISMA) 2009 guidelines, and the Cochrane Collaboration definition for systematic review and meta-analysis were adhered to. A search was systematically done in the PubMed, Embase, Web of Sciences, and Cochrane Library databases for relevant studies conducted between June 1937 and April 2020. The search strategy used were “Parkinson”, OR “Parkinson'”, OR “Parkinson's”, OR “Parkinson disease”, OR “Parkinson' disease”, OR “Parkinson's disease”, OR “parkinsonism”, OR “parkinsonian”, OR “Multiple System Atrophy”, OR “MSA”) AND (“biomarker”, OR “Cerebrospinal Fluid”, OR “CSF”).

### 2.2. Selection Criteria

Studies included in the meta-analysis were (1) retrospective, prospective cohort, and cross-sectional studies; (2) studies whose participants were diagnosed as having idiopathic PD or with probable/possible MSA; and (3) studies that cerebrospinal fluid biomarkers of PD and MSA were detected. Studies were excluded if they were (1) nonhuman or nonoriginal studies, (2) studies in which the level of CSF biomarkers were not quantified, and (3) nonoriginal research (reviews, meta-analyses, commentaries, letters, reports, conference abstracts, and editorials) and duplicated studies.

### 2.3. Data Extraction and Quality Assessment

Two investigators independently reviewed the retrieved articles to determine their eligibility and extract study data. Data extracted included name of authors, year of publication, sample size, gender composition, mean age, diagnosis criteria, the mean and standard deviation of CSF biomarkers, and assay type. Discrepancies were resolved through discussions and consultations with a third investigator when necessary.

### 2.4. Statistical Analysis

STATA software version 15.0 was applied for all statistical analyses. The majority of the effect sizes (ESs) were generated using the sample size, mean CSF biomarker concentration, and standard deviation (SD). The rest of the ESs were calculated using the median and interquartile range (IQR) if the mean and SD were not reported. The statistical difference of the pooled ES was estimated using a 95% confidence interval (95% CI). On the one hand, a fixed-effects model was used to meta-analyze data showing homogeneity and low heterogeneity. On the other hand, a random-effects model was used to analyze data showing moderate or high heterogeneity.

*I* ^2^ index was used to determine the inconsistency across studies to determine the level of heterogeneity. An *I*^2^ index of 0.25, 0.50, and 0.75 indicated small, medium, and high levels of heterogeneity, respectively. Sensitivity analysis was done by removing one study at a time to test if the outcomes of the meta-analysis were significantly influenced by a single study. Unrestricted maximum-likelihood random-effects metaregression of ESs was used to evaluate if the mean age and gender (proportion of male) were confounders that affected the ESs. Egger's test was used to assess publication bias. A *P* value of less than 0.05 (*P* < 0.05) was considered statistically significant.

## 3. Results and Discussion

**Figure 1:**
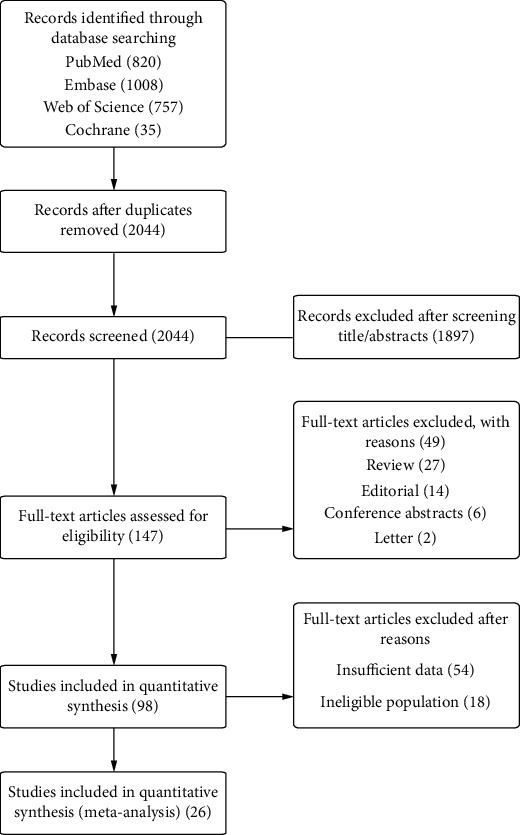
Flow diagram of systematic literature searching.

The literature search yielded 2620 potentially relevant articles (Figure 1). 2044 articles were reviewed after eliminating duplicates. 147 articles were identified for full-text scrutiny after the screening of titles and abstracts. 121 were excluded because they were either reviews (*n* = 27), editorials (*n* = 14), letters (*n* = 2), conference abstracts (*n* = 6), having ineligible population (*n* = 18), or having insufficient data (*n* = 54). The remaining 26 studies [18–43] were included in the meta-analysis (Supplementary Table 1,1). They comprised 2597 unique participants: 1803 PD patients and 794 MSA patients.

## 4. Association of PD and MSA with Pathological Marker

**Figure 2:**
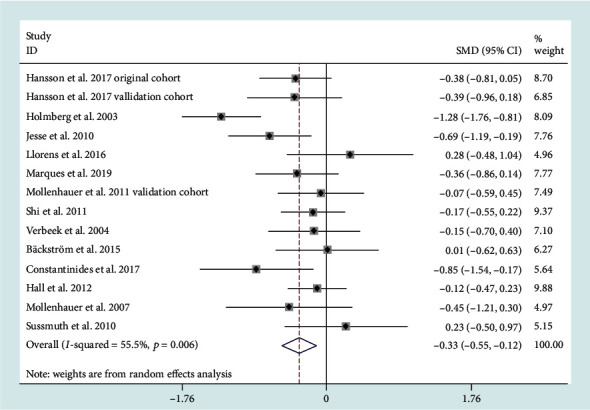
Cerebrospinal fluid (CSF) levels of A*β*42 in multiple system atrophy (MSA) cohorts were lower than those in Parkinson's disease (PD) cohorts. SMD: standard mean difference; CI: confidence interval.

15 studies comprising 1065 PD patients and 322 MSA patients reported decreased CSF amyloid-beta 1-42 (A*β*-42) levels (SMD = −0.33, 95% CI: -0.55 to -0.12) in MSA patients compared to the PD patients (Figure 2). The heterogeneity was medium (*I*^2^ = 55.5%). Subgroup and sensitivity analyses were unable to reveal the source of heterogeneity. A metaregression analysis of age and gender indicated that both variables had no effects on the outcome.

## 5. Association of PD and MSA with Neuronal Injury Markers

**Figure 3:**
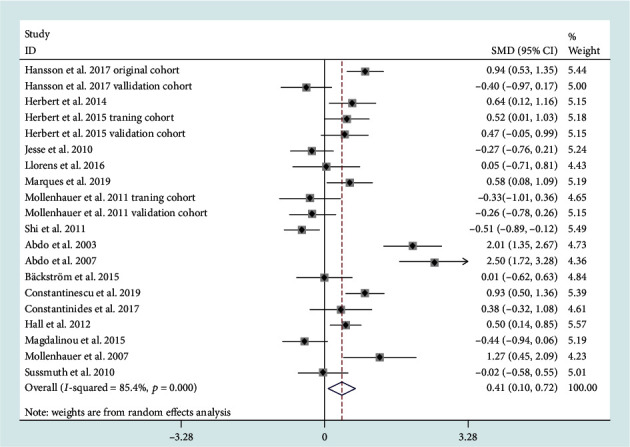
Cerebrospinal fluid (CSF) levels of total microtubule-associated protein (t-tau) in multiple system atrophy (MSA) cohorts were higher than those in Parkinson's disease (PD) cohorts.

19 studies reported CSF total microtubule-associated protein (t-tau) levels in patients diagnosed with PD and MSA. Random-effects meta-analysis demonstrated that CSF t-tau levels (SMD = 0.41, 95% CI: 0.10 to 0.72) in MSA patients were significantly higher compared to those in PD patients (Figure 3). However, the heterogeneity was high (*I*^2^ = 85.4%). Subgroup analysis based on assay type did not reduce the heterogeneity. Heterogeneity in CSF t-tau levels between PD and MSA patients was not influenced by any study based on sensitivity analysis. Metaregression analysis on age and gender revealed that there was a significant association between age and ES (regression coefficient = −0.12, 95% CI: −0.18 to −0.06, *P* < 0.001). As such, age had moderating effects on the outcomes of the meta-analysis.

**Figure 4:**
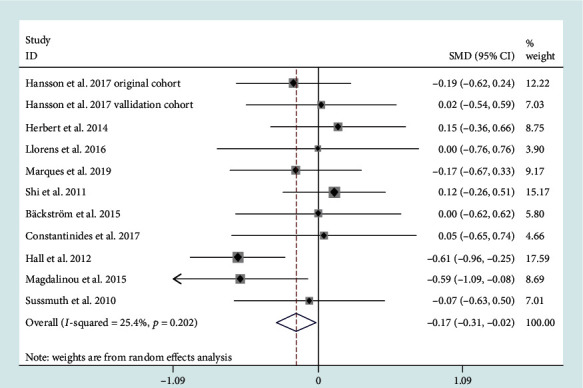
Cerebrospinal fluid (CSF) levels of phosphorylated tau (p-tau) in multiple system atrophy (MSA) cohorts were lower than those in Parkinson's disease (PD) cohorts after sensitivity analysis.

A meta-analysis of 14 studies revealed that CSF phosphorylated tau (p-tau) levels had no difference between PD and MSA patients (SMD = −0.05, 95% CI: -0.28 to 0.17) (Figure S1). They had medium heterogeneity (*I*^2^ = 59.9%). Sensitivity analysis revealed that a study conducted by Constantinescu et al. [27] was the main source of heterogeneity. After eliminating the study, CSF p-tau levels in MSA patients were lower than those in PD patients (SMD = −0.17, 95% CI: -0.31 to -0.02) (Figure 4). The heterogeneity was also reduced (*I*^2^ = 25.4%).

Three original studies were available for meta-analysis of CSF glial fibrillary acidic protein (GFAP) levels. There were no differences in CSF GFAP levels between PD and MSA patients (SMD = 0.20, CI: -0.21 to 0.60) (Figure S2). No heterogeneity existed among the studies (*I*^2^ = 0).

## 6. Association of PD and MSA with Neuroinflammation Markers

**Figure 5:**
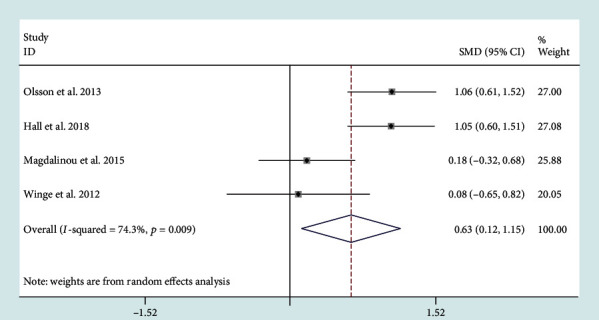
Cerebrospinal fluid (CSF) levels of YKL-40 in multiple system atrophy (MSA) cohorts were higher than those in Parkinson's disease (PD) cohorts.

Four studies reported CSF YKL-40 levels. CSF YKL-40 levels (SMD = 0.63, 95% CI: 0.12 to 1.15) were significantly higher in MSA patients compared to PD patients (Figure 5). Their heterogeneity was medium (*I*^2^ = 74.3%).

A meta-analysis of CSF fms-related tyrosine kinase 3 ligand (Flt3 ligand) revealed that MSA and PD patients had similar levels of Flt3 ligand (SMD = −0.50, 95% CI: -1.48 to 0.48) (Figure S3). However, their heterogeneity was high (*I*^2^ = 94.8%). One study [24] was identified as the source of heterogeneity based on sensitivity analysis. After removing the study from the analysis, the heterogeneity disappeared (*I*^2^ = 0%) and the difference in Flt3 ligand levels did not still exist (SMD = −0.04, 95% CI: -0.29 to 0.21).

## 7. Association of PD and MSA with Oxidative Stress Markers

**Figure 6:**
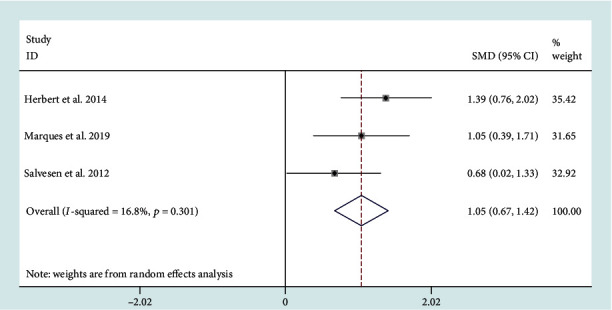
Cerebrospinal fluid (CSF) levels of DJ-1 in multiple system atrophy (MSA) cohorts were higher than those in Parkinson's disease (PD) cohorts.

A meta-analysis of 4 studies revealed CSF DJ-1 levels in both MSA and PD patients which lacked a significant effective size (SMD = 0.61, 95% CI: -0.39 to 1.62) (Figure S4). However, their heterogeneity was high (*I*^2^ = 92%). One study [2] was identified as the source of heterogeneity based on sensitivity analysis. After removing the study from the analysis, the impact of heterogeneity was reduced to 16.8% and the difference in CSF DJ-1 levels between PD and MSA patients appeared (SMD = 1.05, 95% CI: 0.67 to 1.42) (Figure 6).

## 8. Publication Bias

Egger's test revealed that there was no significant risk of publication bias observed among the included studies (Supplementary table 2).

## 9. Discussion

To the author's knowledge, this study is the first to pool data from studies evaluating CSF biomarkers associated with MSA and PD. CSF t-tau, YKL-40, and DJ-1 levels were higher in MSA patients than in PD patients while CSF p-tau and A*β*-42 levels were lower in MSA patients than in PD patients. CSF GFAP and Flt3 ligand levels were similar in both MSA and PD patients.

A*β*-42 is a 42 amino-acid long aggregation-prone protein marker of A*β* plaque pathology. In most studies, A*β*42 is significantly reduced in PD compared with controls and is associated with worse cognitive performance [8, 44]. Although dementia is a feature not supporting a diagnosis of MSA [2], emerging evidence has demonstrated that cognitive impairments are an integral part of the disease [45, 46]. In our study, reduced CSF A*β*-42 concentration implied a cognitive deficiency in the MSA cohort. NF-L is a promising marker for differential diagnosis of parkinsonism. It is essential in maintaining the axonal caliber as well as the neuronal shape and size [8]. Similarly, tau is involved in microtubule assembly and stabilization. It also plays an important role in the structural integrity of the neuron and axonal support [47]. Accumulation of abnormal tau in neurons and glial cells is the main contributing factor to neurodegeneration in Alzheimer's disease (AD), corticobasal degeneration (CBD), and PSP patients [47]. Compared with PD patients, elevated CSF t-tau and NF-L concentration in MSA patients revealed that MSA patients suffered from more pronounced axonal degeneration. This was an indication of underlying severer clinical manifestations of MSA. In our study, CSF level of p-tau was lower in MSA patients than it was in PD patients, which reflects the difference of the phosphorylation state of tau and the formation of neurofibrillary tangles in those two diseases [48]. YKL-40, highly expressed in astrocytes and microglia [49], has been reported to contribute to neuroinflammation [50]. In our study, CSF YKL-40 level was higher in MSA patients than it was in PD patients, which reflected increased glial activation in MSA patients and implied that more serious neuroinflammation could be involved in MSA pathogenesis compared with PD patients. DJ-1 is encoded by the PARK7 gene. It has a protective antioxidant role in reactive astrocyte [51]. Higher DJ-1 levels in MSA patients compared with PD patients may be a compensatory neuroprotective mechanism to excessive oxidative stress. Although this study did not show any discrepancyies in CSF's GFAP and Flt3 ligand between PD and MSA patients, the results were inconclusive because of a limited number of original studies.

The heterogeneity among studies varied from zero to high. The heterogeneity of CSF t-tau and YKL-40 levels was significantly high. A subgroup analysis of the array type could not reduce it. Cognizant of this, we speculated that antibody characteristics, protocols of lumbar puncture, and the quality of CSF samples were responsible for discrepant results. As for both Flt3 ligand and DJ-1, the heterogeneity could be derived from the study conducted by Shi et al. [24]. The possible reasons are the small sample size of MSA patients and the different medications used in MSA and PD patients of this study.

Nevertheless, this study was limited by several factors. One study demonstrated that elevated CSFA*β*-42 and tau concentration was associated with cognitive dysfunction in PD cohorts [52]. However, our metaregression analyses revealed that age was a confounding factor affecting the outcome of the meta-analysis. Whether other clinical variables like disease duration, disease severity, and medication affected the result in this study was unclear. The included studies did not provide detailed clinical information thus hindering further analyses. The study was also limited by the availability of only a few original studies with the deficient sample size for meta-analysis of GFAP and Flt3 ligand. This might have caused false negatives. Moreover, in all the included studies, patients were diagnosed based on clinical manifestations without pathologic confirmation. As such, the diagnostic accuracy was unclear. Finally, although our study revealed there are differences in several CSF biomarker levels between patients with MSA and PD, the diagnostic value of these biomarkers is still unclear. Cognizant of these, the results of this study should be interpreted with caution.

In the future, researchers should consider the effect of clinical features on the levels of biomarkers. A standard protocol for CSF analysis should also be developed for consistency. Further to this, prospective studies comprising a large number of samples focusing on diverse types of candidate biomarkers are needed for more conclusive results and diagnostic tests are necessary to clarify the validity and reliability of candidate biomarkers. More attention should also be put on combinations of different biomarker modalities as it may provide valuable clues in improving the diagnostic accuracy of PD and MSA.

## 10. Conclusions

CSF t-tau, YKL-40, and DJ-1 levels were higher in MSA patients than in PD patients. Age partially accounted for the heterogeneity. There were alterations of CSF biomarker levels between MSA and PD patients though longitudinal studies comprising a large number of samples are necessary to clarify this issue. Detailed clinical features and combination of biomarkers should be considered in future studies. Nonetheless, this study revealed the differences in several CSF biomarker levels between MSA and PD cohorts that can be further exploited to clinically distinguish MSA from PD.

## Data Availability

No data were used to support this study.

## Supplementary Materials

Supplementary 1 Figure S1: CSF phosphorylated tau (p-tau) levels had no difference between PD and MSA patients (SMD = −0.05, 95% CI: -0.28 to 0.17).

Supplementary 2 Figure S2: there were no differences in CSF GFAP levels between PD and MSA patients (SMD = 0.20, CI: -0.21 to 0.60).

Supplementary 3 Figure S3: a meta-analysis of CSF fms-related tyrosine kinase 3 ligand (Flt3 ligand) revealed that MSA and PD patients had similar levels of Flt3 ligand (SMD = −0.50, 95% CI: -1.48 to 0.48).

Supplementary 4 Figure S4: through meta-analysis of 4 studies, CSF DJ-1 levels in both MSA and PD patients lacked a significant effective size (SMD = 0.61, 95% CI: -0.39 to 1.62).

Supplementary 5 Supplementary Table 1: characteristics of studies included in the meta-analyses.

Supplementary 6 Supplementary Table 2: summary of Egger's test outcomes for measurements of CSF biomarker levels.
