# Supplementary material for: Cerebrospinal Fluid Biomarkers in Multiple System Atrophy Relative to Parkinson's Disease: A Meta-Analysis
Source: Behav Neurol. 2021 May 31;2021:5559383. doi: 10.1155/2021/5559383 (PMC8188602; doi:10.1155/2021/5559383)
Supplement: Supplementary 1 — Figure S1: CSF phosphorylated tau (p-tau) levels had no difference between PD and MSA patients (SMD = −0.05, 95% CI: -0.28 to 0.17). [file 5559383.f1.docx]

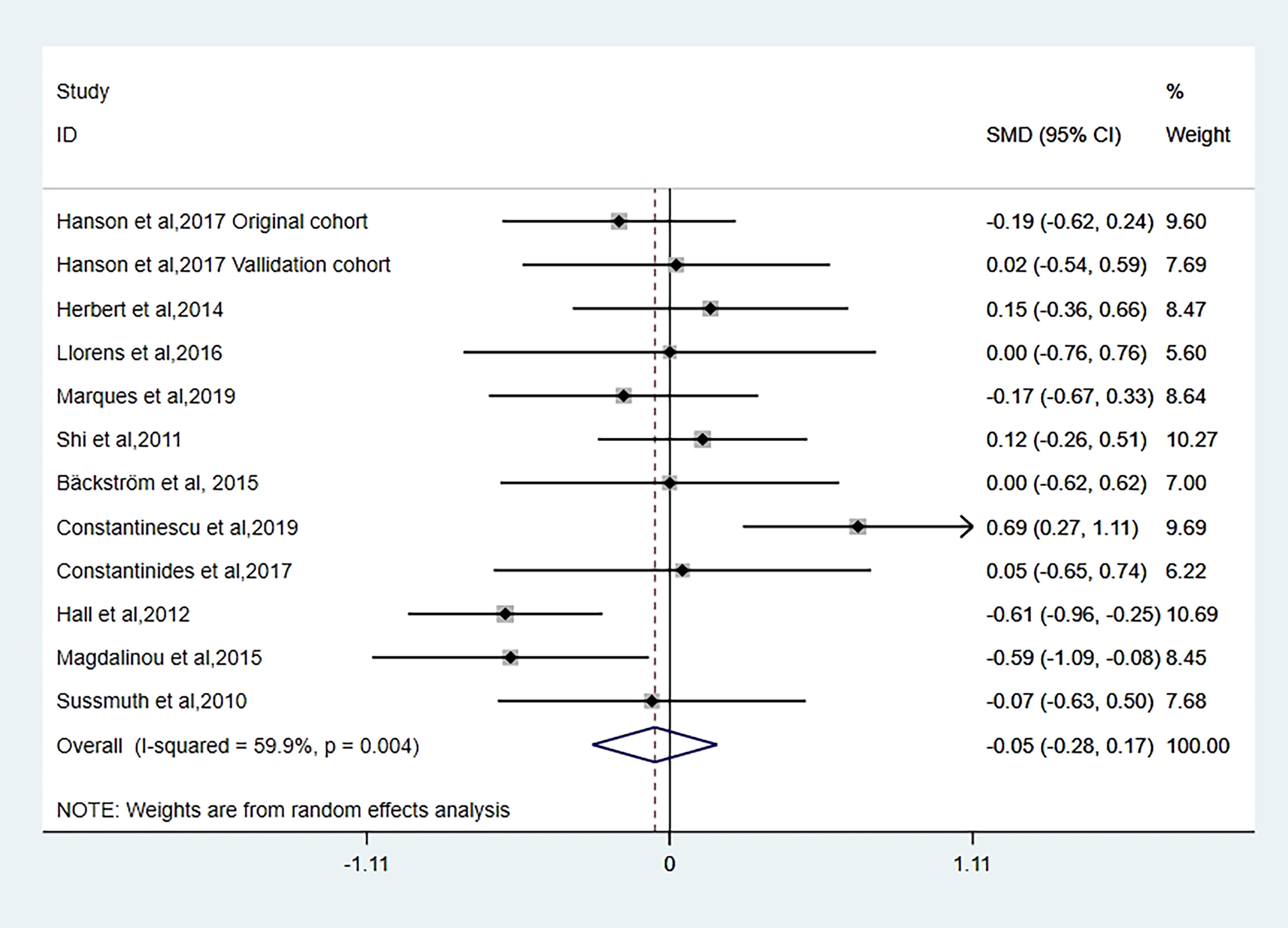


Figure S1: Cerebrospinal fluid (CSF) levels of phosphorylated tau (p-tau) in Multiple system atrophy (MSA) cohorts had no difference from that in Parkinson’s disease (PD) cohorts.
