# Supplementary material for: Cerebrospinal Fluid Biomarkers in Multiple System Atrophy Relative to Parkinson's Disease: A Meta-Analysis
Source: Behav Neurol. 2021 May 31;2021:5559383. doi: 10.1155/2021/5559383 (PMC8188602; doi:10.1155/2021/5559383)
Supplement: Supplementary 2 — Figure S2: there were no differences in CSF GFAP levels between PD and MSA patients (SMD = 0.20, CI: -0.21 to 0.60). [file 5559383.f2.docx]

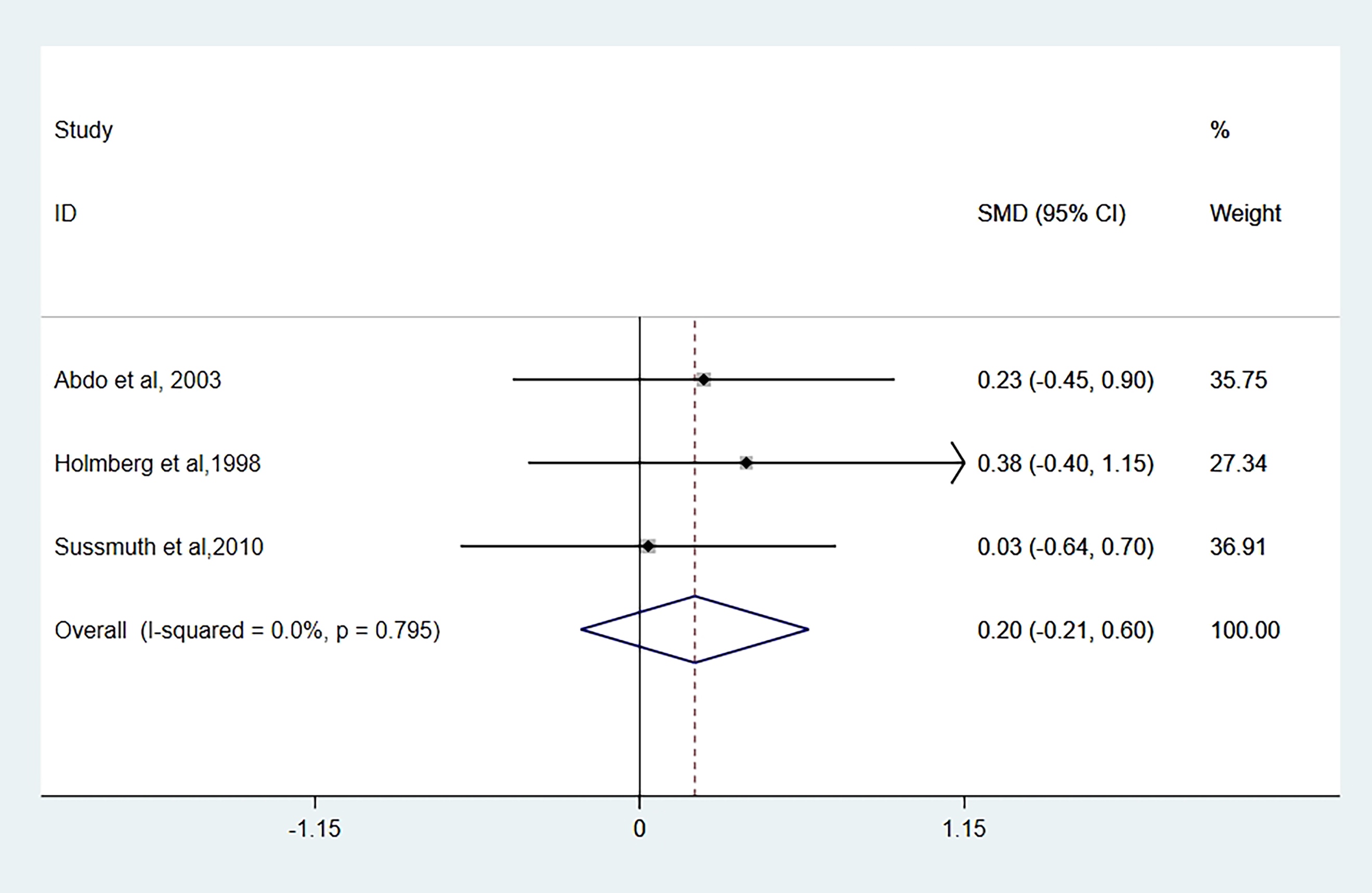


Figure S2: Cerebrospinal fluid (CSF) levels of glial fibrillary acidic protein (GFAP) in Multiple system atrophy (MSA) cohorts had no difference from that in Parkinson’s disease (PD) cohorts.
