# Supplementary material for: Cerebrospinal Fluid Biomarkers in Multiple System Atrophy Relative to Parkinson's Disease: A Meta-Analysis
Source: Behav Neurol. 2021 May 31;2021:5559383. doi: 10.1155/2021/5559383 (PMC8188602; doi:10.1155/2021/5559383)
Supplement: Supplementary 3 — Figure S3: a meta-analysis of CSF fms-related tyrosine kinase 3 ligand (Flt3 ligand) revealed that MSA and PD patients had similar levels of Flt3 ligand (SMD = −0.50, 95% CI: -1.48 to 0.48). [file 5559383.f3.docx]

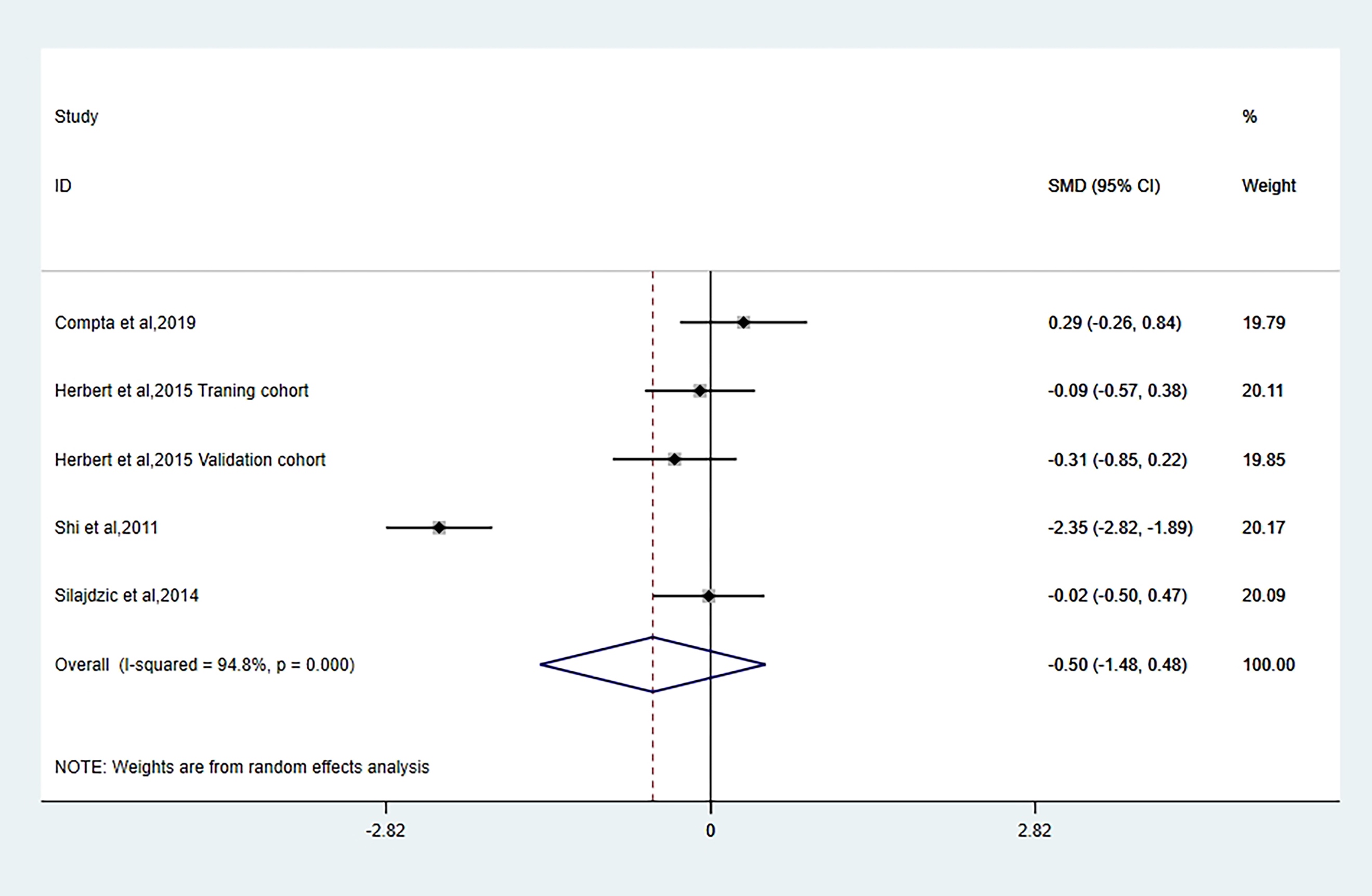


Figure S3: Cerebrospinal fluid (CSF) levels of fms-related tyrosine kinase 3 ligand (Flt3 ligand) in Multiple system atrophy (MSA) cohorts had no difference from that in Parkinson’s disease (PD) cohorts.
