# Supplementary material for: Cerebrospinal Fluid Biomarkers in Multiple System Atrophy Relative to Parkinson's Disease: A Meta-Analysis
Source: Behav Neurol. 2021 May 31;2021:5559383. doi: 10.1155/2021/5559383 (PMC8188602; doi:10.1155/2021/5559383)
Supplement: Supplementary 4 — Figure S4: through meta-analysis of 4 studies, CSF DJ-1 levels in both MSA and PD patients lacked a significant effective size (SMD = 0.61, 95% CI: -0.39 to 1.62). [file 5559383.f4.docx]

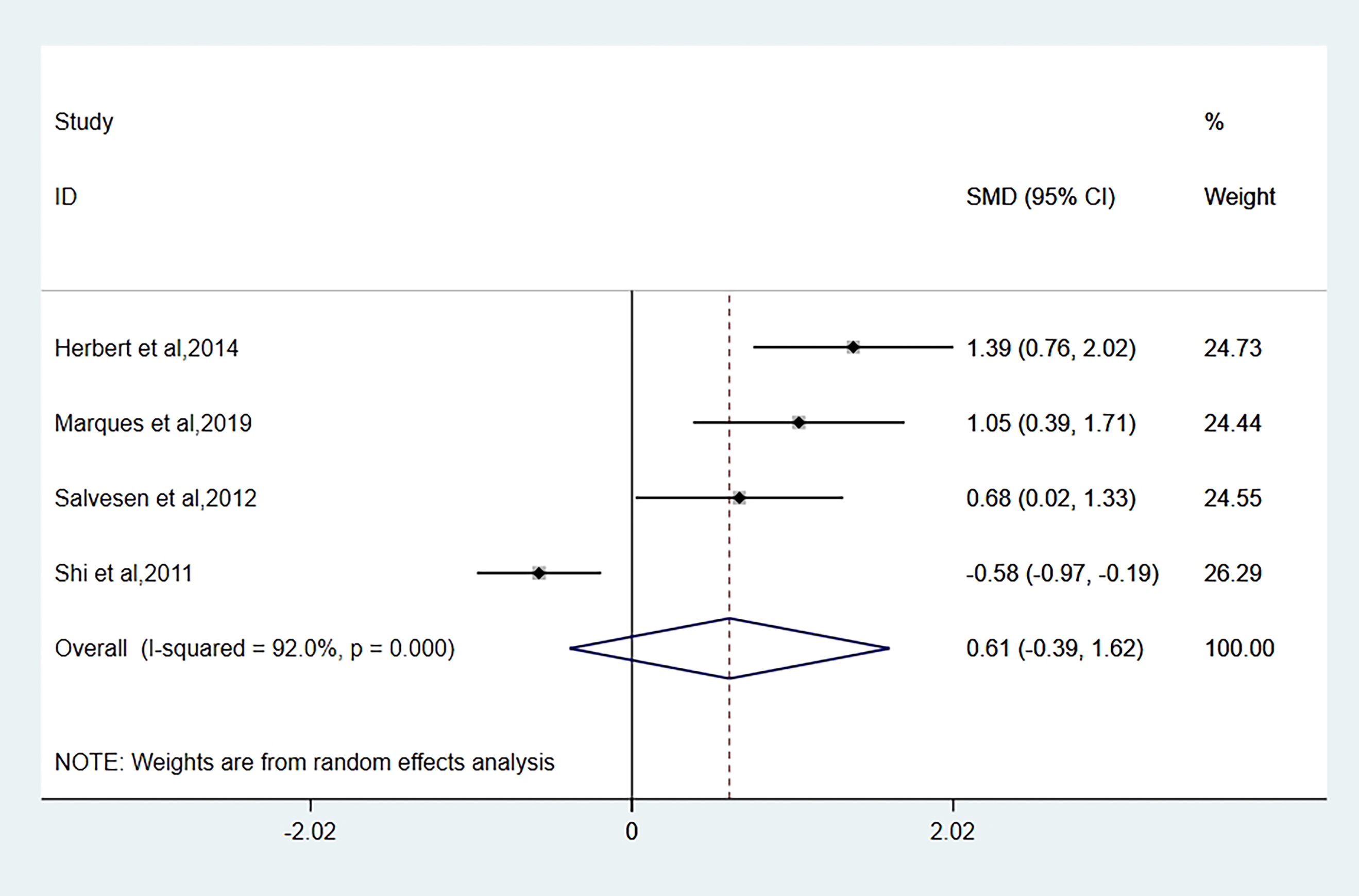


Figure S4: Cerebrospinal fluid (CSF) levels of DJ-1 in Multiple system atrophy (MSA) cohorts had no difference from that in Parkinson’s disease (PD) cohorts.
