# Supplementary material for: Cerebrospinal Fluid Biomarkers in Multiple System Atrophy Relative to Parkinson's Disease: A Meta-Analysis
Source: Behav Neurol. 2021 May 31;2021:5559383. doi: 10.1155/2021/5559383 (PMC8188602; doi:10.1155/2021/5559383)
Supplement: Supplementary 5 — Supplementary Table 1: characteristics of studies included in the meta-analyses. [file 5559383.f5.docx]

Supplementary Table 1. Characteristics of studies included in the meta-analyses.

| Study/Year | Country | Sample size  (PD/MSA) | Gender (male %)  (PD/MSA) | Mean age (PD/MSA) | Diagnosis | Assay type | medication |
| --- | --- | --- | --- | --- | --- | --- | --- |
| Abdo et al, 2003 | The Netherlands | 35/29 | 60/56.4 | 53/61.5 | PD: PDSBB MSA: Gilman et al,1999[1] | ELISA | Y |
| Abdo et al, 2007 | The Netherlands | 31/19 | NA | 52.5/59.6 | PD:PDSBB MSA: Litvan et al, 2003[2] | ELISA | NA |
| Bäckström et al,2015 | Sweden | 99/11 | 58.6/72.7 | 71.3/72.9 | PD: PDSBB MSA: Gilman et al,1999[1] | ELISA | NA |
| Compta et al, 2019 | Spain | 19/39 | 63/56 | 58/57 | PD: PDSBB MSA: Gilman et al,2008[3] | Luminex | Y |
| Constantinescu et al,2019 | Sweden | 68/34 | 67.6/41 | 63/65 | PD: PDSBB MSA: Gilman et al,2008[3] | ELISA | NA |
| Constantinides et al,2017 | Greece | 17/15 | 58.8/66.7 | 58.2/63.4 | PD: PDSBB MSA: Gilman et al,2008[3] | ELISA | Y |
| Hall et al,2012 | Sweden | 90/48 | 67/46 | 63/64 | PD: Gelb et al.1999[4] MSA: Gilman et al,1999[1] | Luminex | NA |
| Hall et al,2018 | Sweden | 131/24 | 61/50 | 64.9/71.5 | PD: Gelb et al.1999[4] MSA: Gilman et al,1999[1] | ELISA | NA |
| Hanson et al,2017 | Sweden | 171/30 | 63/57 | 65/64 | PD: Gelb et al.1999[4] MSA: Gilman et al,1999[1] | ELISA | NA |
| Herbert et al,2014 | The Netherlands | 43/23 | 67/61 | 58.9/60.5 | PD: PDSBB MSA: Gilman et al,2008[3] | ELISA | NA |
| Herbert et al,2015 | The Netherlands | 68/59 | 66/48 | 58.4/62 | PD: PDSBB MSA: Gilman et al,2008[3] | ELISA | NA |
| Holmberg et al,1998 | Sweden | 19/10 | 58/50 | 64.9/63.7 | PD: PDSBB MSA: Quinn et al,1989[5] | ELISA | NA |
| Holmberg et al,2003 | Sweden | 48/36 | 58/53 | 62.3/63.7 | PD: PDSBB MSA: Quinn et al,1989[5] | ELISA | NA |
| Jesse et al,2010 | Germany | 47\25 | NA | 66/65 | NA | ELISA | NA |
| Llorens et al,2016 | Germany | 40\11 | NA | 66/66 | PD: PDSBB MSA: Gilman et al,2008[3] | ECL-based ELISA | NA |
| Magdalinou et al,2015 | Sweden | 31\31 | 64.5/51.6 | 67.1/64.3 | PD: PDSBB MSA: Gilman et al,2008[3] | ELISA | NA |
| Marques et al,2019 | The Netherlands | 55/22 | 69/68 | 57/60.7 | PD: PDSBB MSA: Gilman et al,2008[3] | ELISA | Y |
| Mollenhauer et al,2007 | America | 11/18 | 73/56 | 70/62 | PD: PDSBB MSA: Gilman et al,1999[1] | ELISA | NA |
| Mollenhauer et al,2011 | America | 324/44 | 67/59 | 72.2/68 | PD: PDSBB MSA: Gilman et al,2008[3] | ELISA | Y |
| Olsson et al,2013 | Sweden | 50/32 | 72/37.8 | 60.4/64.6 | PD: Gelb et al.1999[4] MSA: Gilman et al,1999[1] | ELISA | NA |
| Salvesen et al,2012 | Denmark | 30/14 | 63/50 | 57/59.5 | NA | ELISA | NA |
| Shi et al,2011 | America | 137/32 | 55/66 | 63.8/60.3 | PD: PDSBB MSA: Gilman et al,2008[3] | Luminex | NA |
| Silajdzic et al,2014 | Sweden | 37/30 | 78/40 | 60.7/64.1 | PD: Gelb et al.1999[4] MSA: Gilman et al,1999[1] | ELISA | NA |
| Sussmuth et al,2010 | Germany | 23/25 | 57/64 | 66.1/62.5 | PD: Gelb et al.1999[4] MSA: Gilman et al,1999[1] | Luminex | Y |
| Verbeek et al,2004 | The Netherland | 30/22 | 60/77 | 52.8/59.8 | PD: PDSBB MSA: Gilman et al,1999[1] | ELISA | NA |
| Winge et al,2010 | Denmark | 24/10 | NA | NA | NA | ELISA | NA |

Abbreviation: ELISA: enzyme linked immunosorbent assay; PDSBB: UK Parkinson's Disease Society Brain Bank clinical diagnosis criteria; Y: Yes; NA: Not Available.

**Reference**

[1] S. Gilman, P.A. Low, N. Quinn, A. Albanese, Y. Ben-Shlomo, C.J. Fowler, Kaufmann H, T. Klockgether , A.E. Lang , P.L. Lantos , I. Litvan , C.J. Mathias , E. Oliver , D. Robertson , I. Schatz , Wenning GK (1999) Consensus statement on the diagnosis of multiple system atrophy. *Journal of the Neurological Sciences* **163**, 94-98.

[2] Irene Litvan, Kailash P. Bhatia, David J. Burn, Christopher G. Goetz, Anthony E. Lang, Ian McKeith, Niall Quinn, Kapil D. Sethi, Cliff Shults, Wenning GK (2003) SIC Task Force Appraisal of Clinical Diagnostic Criteria for Parkinsonian Disorders. *Mov Disord* **18**, 467-486.

[3] S. Gilman GKW, P.A. Low, D.J. Brooks, C.J. Mathias, J.Q. Trojanowski, N.W. Wood, C. Colosimo, A. Durr, C.J. Fowler, H. Kaufmann, T. Klockgether, A. Lees, W. Poewe, N. Quinn, T. Revesz, D. Robertson, P. Sandroni, K. Seppi, M. Vidailhet (2008) Second consensus statement on the diagnosis of MSA. *Neurology* **71**, 670-676.

[4] Douglas J. Gelb EO, Sid Gilman (1999) Diagnostic criteria for Parkinson disease. *Arch Neurol* **56**, 33-39.

[5] Quinn N (1989) Multiple System Atrophy--The Nature of the Beast. *Journal of Neurology, Neurosurgery, and Psychiatry* **Suppl**, 78-89.
