# Supplementary material for: Cerebrospinal Fluid Biomarkers in Multiple System Atrophy Relative to Parkinson's Disease: A Meta-Analysis
Source: Behav Neurol. 2021 May 31;2021:5559383. doi: 10.1155/2021/5559383 (PMC8188602; doi:10.1155/2021/5559383)
Supplement: Supplementary 6 — Supplementary Table 2: summary of Egger's test outcomes for measurements of CSF biomarker levels. [file 5559383.f6.docx]

Supplementary Table 2: Summary of Egger’s test Outcomes for Measurements of CSF biomarker levels.

| CSF biomarker | No. of studies | Publication bias | |
| --- | --- | --- | --- |
|  |  | Egger Intercept | P value |
| Aβ-42 | 15 | -1.38 | 0.303 |
| t-tau | 19 | 0.92 | 0.369 |
| p-tau | 14 | 0.44 | 0.666 |
| GFAP | 3 | 1.66 | 0.345 |
| YKL-40 | 4 | -1.38 | 0.303 |
| Flt3 ligand | 4 | 1.27 | 0.295 |
| DJ-1 | 4 | 4.32 | 0.050 |

Abbreviation: Aβ-42: amyloid beta 1-42; t-tau: total microtubule-associated protein; p-tau: phosphorylated microtubule-associated protein; GFAP: glial fibrillary acidic protein; Flt3 ligand: fms-related tyrosine kinase 3 ligand.
